# Sequence and phylogenetic analysis of a novel alphaendornavirus, the first virus described from the oomycete plant pathogen *Phytophthora heveae*

**DOI:** 10.1007/s00705-023-05786-7

**Published:** 2023-05-11

**Authors:** Milica Raco, Thomas Jung, Marilia Horta Jung, Nguyen Minh Chi, Leticia Botella, Nobuhiro Suzuki

**Affiliations:** 1 Phytophthora Research Centre, Department of Forest Protection and Wildlife Management, Faculty of Forestry and Wood Technology, Mendel University in Brno, Brno, Czech Republic Zemědělská 3, 613 00; 2 Forest Protection Research Centre, Vietnamese Academy of Forest Sciences, Hanoi, 10000 Vietnam; 3 Institute of Plant Science and Resources, Okayama University, Kurashiki, 7100046 Japan Chuo 2-20-1,

## Abstract

Here, we report the discovery and complete genome sequence of a novel virus, designated as “Phytophthora heveae alphaendornavirus 1” (PhAEV1), from a single isolate of the plant pathogenic oomycete *Phytophthora heveae* (kingdom *Stramenipila*) isolated from a tropical evergreen lowland rainforest in northern Vietnam. PhAEV1 was detected by both cellulose affinity chromatography of dsRNA and high-throughput sequencing of total RNA, and its presence and sequence were confirmed by RT-PCR and Sanger sequencing. The PhAEV1 genome, 12,820 nucleotides (nt) in length, was predicted to encode a single large polyprotein with the catalytic core domain of viral (superfamily 1) RNA helicase (HEL, amino acid [aa] positions 1,287-1,531), glycosyltransferase (GT, aa positions ca. 2,800-3,125), and RNA-directed RNA polymerase (RdRp, aa positions 3,875-4,112). PhAEV1 is the most similar to Phytophthora cactorum alphaendornavirus 3, sharing 39.4% and 39.1% nt and aa sequence identity, respectively. In addition to the first 5′-terminal AUG codon, three additional in-frame methionine codons were found in close proximity (nt 14-16, 96-98, and 176-178), suggesting potential additional translation initiation sites. Conserved RdRp motifs (A-E) similar to those detected in related endornaviruses were identified in PhAEV1, as well as in several previously described alphaendornaviruses from other *Phytophthora* species in which these motifs had not been identified previously. Phylogenetic analysis showed that PhAEV1 clusters with members of the genus *Alphaendornavirus* in the family *Endornaviridae* and is basal to two other alphaendornaviruses described from another oomycete, *Phytophthora cactorum*. PhAEV1 is the first virus reported in *P. heveae*.

**Supplementary Information:**

The online version contains supplementary material available at 10.1007/s00705-023-05786-7.

*Phytophthora heveae* (A.W. Thomps.) is a plant-pathogenic homothallic oomycete (*Stramenipila* (syn. Heterokonta), *Oomycota*, *Peronosporales*, *Peronosporaceae*) that is placed taxonomically in phylogenetic major clade 5 of the genus *Phytophthora*. It is known as a root and bark pathogen of *Theobroma* L., *Persea* Mill. and *Rhododendron* L., distributed across East and Southeast Asia; Central, North, and South America; and Oceania [1–7]. The genus *Phytophthora* is entirely composed of plant pathogens and includes some of the most devastating species threatening agricultural, forest, and natural ecosystems worldwide [3, 8]. Mycoviruses, which, by broad definition are fungal viruses and oomycete viruses, generally infect their hosts persistently, in most cases causing cryptic infections with no apparent symptoms. However, they have attracted considerable attention because of their potential to reduce fungal virulence and cause “hypovirulence” [9]. Previously, there have been no records of viral infection in *P. heveae*, although a range of RNA viruses from different families with diverse genome organizations have been described in other *Phytophthora* species [10–17]. Additionally, several ORFan sequences of putative viral origin have been detected in *Phytophthora castaneae* Katsura & K. Uchida [10], which belongs to the same phylogenetic clade (clade 5) as *P. heveae* [6]. The family *Endornaviridae* includes linear positive (+) sense, single-stranded (ss) RNA viruses that do not form true virions. Although previously classified as double-stranded (ds) RNA viruses, their dsRNA form is now considered a replicative intermediate that accumulates during replication of the ssRNA genome [18]. Their genome contains a single large open reading frame (ORF) encoding a polyprotein ranging in size from 3,217 to 5,825 amino acids (aa) with a highly conserved RNA-directed RNA polymerase (RdRp) domain and an RNA helicase domain located in the C-terminal and middle region, respectively. Some endornaviruses encode other functional domains, including methyltransferase, glycosyl transferase, cysteine-rich, phytoreo_S7, and capsular polysaccharide synthase domains [18, 19]. The family is divided into two genera: *Alphaendornavirus,* which includes viruses that infect plants, fungi, and oomycetes [19], and *Betaendornavirus*, which includes viruses that infect ascomycetous fungi [20]. A characteristic that is common to many alphaendornaviruses is the occurrence of a site-specific break (nick) located at a 5’-terminal end of the coding strand [18], the biological significance of which is unknown. Alphaendornaviruses have been reported in several members of the genus *Phytophthora*, including the *Phytophthora* taxon “douglas-fir” [21], *P. ramorum* Werres, De Cock & Man in ’t Veld [22], *P. cactorum* [13], a Vietnamese isolate of *P. castaneae* [10], and *Phytophthora* spp. infecting asparagus in Japan [15]. Compared to viruses of ascomycetes or basidiomycetes, viruses infecting oomycetes, including members of the genus *Phytophthora,* have not been extensively studied, and overall, our knowledge of the effects of viruses on their *Phytophthora* hosts is limited [23]. Plant and fungal endornaviruses are usually associated with asymptomatic infections, and only a few viruses are associated with phenotypic alterations. Vicia faba endornavirus VfEV is associated with cytoplasmatic male sterility traits in the broad bean *Vicia faba* L., while Helicobasidium mompa endornavirus 1 (HmEV1) infecting a basidiomycete *Helicobasidium mompa* Nobuj. Tanaka 1891, appears to confer hypovirulence to its host [19]. Infection by Rhizoctonia solani endornavirus 1 (RsEV1), the putative member of the family *Endornaviridae* with the largest genome reported to date, causes metabolic changes in its host *Rhizoctonia solani* Kühn, resulting in hypovirulence [24]. Additionally, Japanese isolates of *Phytophthora* sp. coinfected with two endornaviruses have been reported to exhibit slower growth and reduced hyphal density while producing abundant zoosporangia. Endornaviruses can also alter the fungicide sensitivity of their oomycete host [15].

Here, we report the discovery of a novel alphaendornavirus, the first virus to be described in the oomycetous plant pathogen *P. heveae*.

## Provenance of the virus material

*P. heveae* isolate VN787 was originally isolated in the spring of 2016 from a mixed rhizosphere soil sample of two native *Saraca dives* Pierre, Fl. Forest. Cochinch. t. 386 B. (1898) (Fabaceae) trees growing in a tropical evergreen lowland rainforest in Cuc-Phuong National Park in northern Vietnam (Supplementary Table S1). The isolate was obtained from the culture collection of T. Jung hosted at the Phytophthora Research Centre (PRC), Mendel University in Brno, and was identified based on molecular sequences of the internal transcribed spacer (ITS1-5.8S-ITS2) region of the ribosomal DNA and the cytochrome c oxidase subunit 1 mitochondrial gene (*cox*1), and also based on microscopy of its morphological features [2]. VN787 was maintained on V8-juice agar (V8A; 100 ml of V8 juice [Hermann Pfanner Getränke, Lauterach, Austria], 16 g of agar [Sigma-Aldrich, St. Louis, MO, USA], 2 g of CaCO_3_, and 900 ml of distilled water per liter) at 20-23 °C in the dark. For extractions of total RNA and dsRNA, mycelia were grown on orange serum agar (OSA) (HiMedia Laboratories, Kennett Square, PA, USA) covered with a cellophane membrane (EJA08-100; Gel Company Inc., San Francisco, CA, United States). dsRNA was isolated from approximately 150 mg of mycelia using a method based on cellulose affinity chromatography as described by Suzuki et al. [25] and visualized in a 1% agarose gel after electrophoresis at 50 V for 60 min and staining with ethidium bromide. Total RNA extraction and cDNA synthesis were performed using RNAzol® RT (Sigma-Aldrich, Steinheim, Germany) following the manufacturer’s recommendations with some modifications, and a High-Capacity cDNA Reverse Transcription Kit (Applied Biosciences, Park Ave, NY, USA), respectively, and the quality and/or quantity of the preparations were estimated as described previously [10]. The total RNA of VN787 was sent to SEQme s.r.o (Dobris, Czech Republic) for RNA library construction and high-throughput sequencing (HTS) in a pool with eight other *P. heveae* isolates from Vietnam (Supplementary Table S1), whose total RNA was prepared as described above. Ribosomal RNA (rRNA) was depleted using an NEBNext rRNA Depletion Kit (Human/Mouse/Rat) (New England Biolabs (NEB) Ipswich, MA, USA), and the library was constructed using an NEBNext Ultra II Directional RNA Library Prep Kit for Illumina (NEB). The library was sequenced in paired-end (2 × 150 base-pairs [bp]) mode on an Illumina NovaSeq6000 (DS-150) platform using a NovaSeq S4 v1.5 reagent kit, together with a control library PhiX Control Kit v3 (Illumina). The quality was inspected using the FastQC-0.10.1 program [26] and the reads were assembled *de novo* using Trinity v2.6.5 [27]. The Trinity contigs were compared to a custom virus protein database using the BLASTx (BLASTX 2.10.0+) algorithm [28] with the *E*-value set to 10^−5^. All sequences showing significant similarity to known viruses were aligned in BLASTn (NCBI BLAST+ 2.12.0) to eliminate contigs potentially originating from the host or contaminants. Finally, contigs of interest were imported into Geneious Prime® 2023.0.4 (Biomatters, Auckland, New Zealand) for further manipulation, including further assembly using Geneious *de novo* assembler, specifying medium-low sensitivity. Virus-specific primers used for virus detection, identification of the ends of viral sequences, and confirmation of internal regions where the contigs overlapped or showed signs of variability were designed using Primer 3 2.3.7 in Geneious Prime® 2020.2.3 (Supplementary Table S2). The depth of coverage was calculated using Bowtie 2 v2.3.0 [29] as described by Raco et al. [10]. The 5′- and 3′-terminal sequences of the dsRNA segment were determined by performing RNA-ligase-mediated rapid amplification of cDNA ends (RLM-RACE) [25] and cloning into pGEM®-T Easy Vector System (Promega, Madison, WI, USA), followed by sequencing of the cloned inserts in both directions by the Sanger method. The NCBI CD-search tool [30] and HHpred server on the MPI Bioinformatics Toolkit website [31] were used to conduct a search for putative conserved domains (both tools last accessed on the 15th of April 2023). Phylogenetic analysis was performed based on RdRP amino acid (aa) and viral helicase domain sequences. Sequences were aligned using MAFFT V7.450 [32] in Geneious Prime® 2023.0.4, and TrimAI v. 1.3 software on the Phylemon 2.0 online platform (http://phylemon2.bioinfo.cipf.es) was used to remove unreliable alignment regions. Bayesian phylogenetic trees were constructed using MrBayes 3.2.6 [33], implemented in Geneious Prime® 2023.0.4.

## Sequence properties

**Fig. 1:**
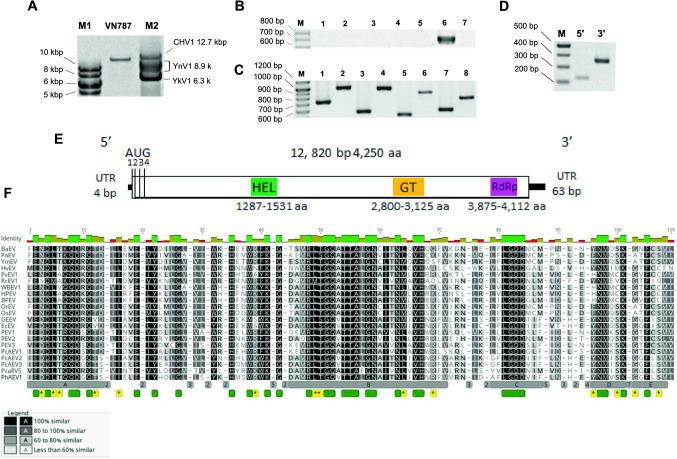
(**A**) dsRNA profile of *P. heveae* isolate VN787. The PhAEV1 replicative form was visualized by ethidium bromide staining in a 1% agarose gel. M1, GeneRuler 1 kb DNA Ladder; M2, dsRNA virus marker consisting of the genomic dsRNA segments or replicative form of Cryphonectria hypovirus 1 (CHV1), yado-nushi virus 1 (YnV1), and yado-kari virus 1 (YkV1), [36, 37]. (**B**) RT-PCR detection of PhAEV1 using the virus-specific primer set PhAEV1_set_1a. Seven out of nine oomycetous isolates used in this study for high-throughput sequencing were tested. Two isolates (VN529 and VN789), whose RNA was pooled for HTS, lost viability during storage. (**C**) Amplification of the PhAEV1 internal sequences by RT-PCR. Virus-specific primer sets (1, PhAEV1_ set_1a; 2, PhAEV1_ set_2; 3, PhAEV1_ set_2a; 4, PhAEV1_ set_3; 5, PhAEV1_ set_3a; 6, PhAEV1_ set_4; 7, PhAEV1_ set_4a; 8, PhAEV1_ set_5; 9, PhAEV1_ set_6) are listed in Supplementary Table S2. (**D**) Amplification of the 5′- and 3′- terminal sequences of the PhAEV1 genomic RNA. RLM-RACE was performed on PhAEV1 dsRNA. Amplified cDNA fragments were analyzed by electrophoresis in a 2% agarose gel. M, 100 bp Plus DNA Ladder (15628019, Thermo Fisher Scientific). (**E**) Schematic representation of the genome structure of PhAEV1. (**F**) Multiple aa sequence alignment of the conserved RdRp region of PhAEV1 and related viruses, performed using MAFFT v7.450 (BLOSUM45 scoring matrix) in Geneious Prime® 2023.0.4. Gray boxes at the bottom of the alignment with letters A-E indicate conserved motifs. Conserved residues are indicated by green boxes and highlighted with a black background, while semi-conserved residues are highlighted in gray. Conserved residues characteristic of alphaendornaviruses that infect *Phytophthora* spp. are indicated by yellow boxes with a star symbol. Numbers with a grey background at the bottom of the alignment indicate the number of residues that were deleted for better visualization. Virus abbreviations: BaEV, Basella alba endornavirus (NC043109); PaEV, Persea americana endornavirus (YP005086952); YmEV, Yerba mate alphaendornavirus (YP009046830); HvEV, Hordeum vulgare alphaendornavirus (YP009212849); PvEV1, Phaseolus vulgaris endornavirus 1 (YP009506353); RcEV1, Rhizoctonia cerealis endornavirus 1 (YP008719905); WBEV1, winged bean alphaendornavirus 1 (YP009305414); HPEV, hot pepper alphaendornavirus (YP009165596); BPEV, bell pepper alphaendornavirus (YP004765011); OrEV, Oryza rufipogon alphaendornavirus (YP438202); OsEV, Oryza sativa alphaendornavirus (YP438200); GEEV, grapevine endophyte alphaendornavirus (YP007003829); EcEV, Erysiphe cichoracearum alphaendornavirus (YP009225663); PEV1, Phytophthora alphaendornavirus 1 (YP241110); PEV2, Phytophthora endornavirus 2 (BCL84886); PEV3, Phytophthora endornavirus 3 (BCL84887); PcAEV1, Phytophthora cactorum alphaendornavirus 1 (QUA12640); PcAEV2, Phytophthora cactorum alphaendornavirus 2 (QUA12641); PcAEV3, Phytophthora cactorum alphaendornavirus 3 (QUA12642); PcaRV5, Phytophthora castaneae RNA virus 5 (USL98317); PhAEV1, Phytophthora heveae alphaendornavirus 1 (OQ786863)

**Fig. 2:**
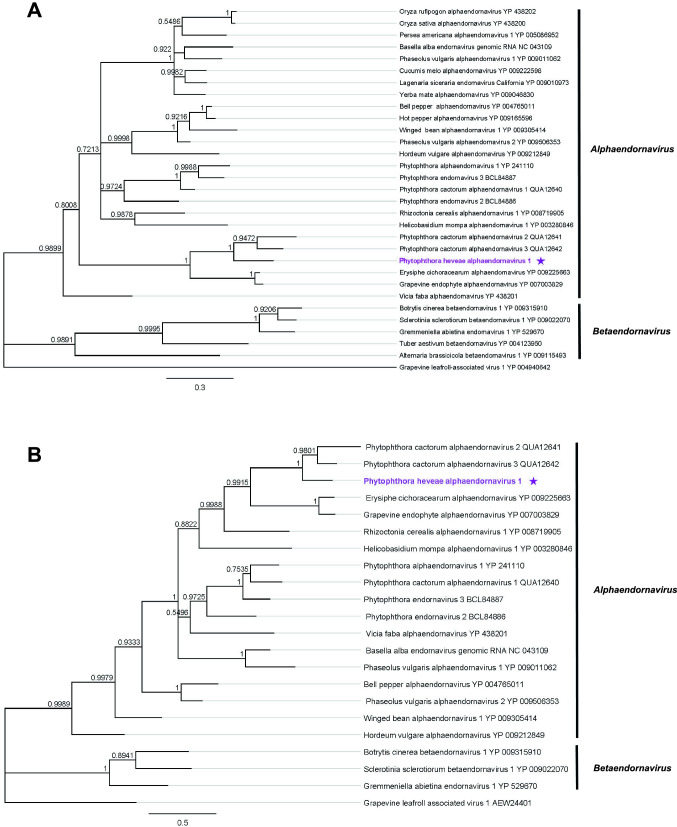
Bayesian trees showing phylogenetic relationships of PhAEV1. The trees were constructed using the aa sequences of the (**A**) RdRp and (**B**) viral helicase domains of PhAEV1 (indicated by a purple star) and those of other endornaviruses. Multiple sequence alignments performed using MAFFT v7.450 (BLOSUM45 scoring matrix) consisted of 201 (**A**) and 175 (**B**) aa sites. The analyses were conducted using MrBayes 3.2.6., including grapevine leafroll-associated virus 1 (genus *Ampelovirus*, family *Closteroviridae*) as an outgroup. Percentage posterior probabilities are displayed at branch nodes. The scale bar shows 0.3 (**A**) and 0.5 (**B**) aa substitutions per site.

Total RNA sequencing generated 509,549,928 reads in total and produced 52,089 Trinity-assembled contigs. BLAST analysis confirmed the presence of multiple virus-like contigs resembling members of several virus families (data not shown), including putative members of the family *Endornaviridae*. The virus characterized here, designated as "Phytophthora heveae alphaendornavirus 1" (PhAEV1), was assembled by Geneious assembler from 20 Trinity contigs ranging in size from 238 bp to 5,262 bp (final contig length obtained by assembly, 12,778 bp, with partial terminal sequences undetermined). We confirmed by dsRNA (Fig. 1A) and RT-PCR analysis (Fig. 1B) that PhAEV1 was present in a single isolate, VN787, but not in the other oomycetous strains used for HTS (Fig. 1B). Virus-specific primers were used to validate regions of overlapping contigs (Fig. 1C), and primer set PhAEV_1_set_1a (Supplementary Table S2) was used for virus detection by RT-PCR in other available cDNAs of the sequencing pool (Fig. 1B). The full-length 12,820-bp-long genomic sequence of PhAEV1, including the 5′-, and 3′-terminal sequences determined by RLM-RACE, was deposited in the GenBank database under accession number OQ786863. Out of the total number of reads, 27,923 mapped to the PhAEV1 sequence. The estimated average depth of coverage for PhAEV1 was 326.71. The 5′-terminal sequence was determined based on 13 individual clones (Fig. 1D). The first nucleotide differed among the clones; five clones had G, four had C, two had T, and two were missing an additional residue. The 3’-terminal sequence was determined based on 10 individual clones that were all identical (Fig. 1D). A single large open reading frame (ORF) of 12,753 nt (4,250 aa) encoding a single polyprotein was detected on the positive (+) strand (frame 2), starting at nt 5 and ending at nt 12,757 (calculated molecular weight, 479.615 kDa) (Fig. 1E). The first methionine codon was observed at nt positions 5-8, but three other in-frame methionine codons were detected in close proximity, 6, 88, and 168, nucleotides downstream from the first AUG, suggesting that these could represent additional translation initiation sites (Fig. 1E). Between the second and third AUG codon, an unusual stretch of five Cs, five Gs, and an A-rich region with 20 A residues was detected. Similar elements have also been observed in Phytophthora cactorum alphaendornavirus 3 (PcAEV3). According to Kozak [34, 35] the optimal sequence for initiation by ribosomal subunits has either a purine base at position nt −3, or a G in position nt +4, or both. Therefore, the second methionine codon with A (-3) and G (+4) occurs in an optimal context for translation initiation. Since it remains to be determined which AUG serves as the main initiation site, the first AUG triplet is tentatively regarded as an initiation codon for PhAEV1. The polyprotein of PhAEV1 encodes a catalytic core domain of RdRp common to members of the family *Endornaviridae* (accession cd23255) at nt 11,627-12,340 (*E*-value, 1.86e-96; CD length, 237 aa), and a viral (superfamily 1) RNA helicase (HEL) domain, which is essential for virus replication, at nt 3,863-4,597 (*E*-value, 6.70e-07; CD length, 227 aa). In addition, a glycosyltransferase (GT) domain was detected using the HHpred tool, but not NCBI CD-search, at the approximate aa position from ~2,800 to 3,125. Five conserved motifs (A-E) were identified by sequence similarity in the RdRp region of PhAEV1 and other related viruses (Fig. 1F). The conserved residues of motifs A-D were fairly similar to those reported previously by Hacker et al. [21]. Additionally, conserved motifs were analyzed in sequences from several other alphaendornaviruses reported previously from *Phytophthora* species for which conserved motifs had not been reported previously (Fig. 1F). According to BLASTx analysis, the nucleotide sequence of PhAEV1 is most similar to the polyprotein sequence of PcAEV3 (QUA12642.1) (query coverage, 95.0%; *E-*value, 0.0; percent identity, 39.4%). At the protein level, the similarity of these two sequences is 39.1% (query coverage, 98.0%; *E-*value, 0.0). PhAEV1 also shows similarity to Krauss’ spikemoss associated endorna-like virus (accession no. CAH2618724.1; query coverage, 87.0%; *E-*value, 0.0; percent identity, 28.4%), and Diatom colony associated dsRNA virus 15 (accession no. YP_009552081.1; query coverage, 90.0%; *E* value, 0.0; percent identity, 27.1%) at the nucleotide level. Phylogenetic analysis showed that PhAEV1 forms a cluster with its descendants, Phytophthora cactorum alphaendornavirus 1 and 2 (PcAEV1 and PcAEV2) (Fig. 2A and B). According to the species demarcation criteria for the genus *Alphaendornavirus* established by the International Committee on Taxonomy of Viruses (ICTV), for viruses to be considered members of different species, they should have an overall nucleotide sequence identity below 75.0%, and their genomes should be greater than 1.9 kb in size [18]. Its phylogeny, genome organization, and sequence divergence support the classification of PhAEV1 as a member of a new species in the genus *Alphaendornavirus*, family *Endornaviridae*.

## Supplementary Information

Below is the link to the electronic supplementary material.

Supplementary file1 (DOCX 24 KB)

Supplementary file2 (FASTA 4 KB)

Supplementary file3 (FASTA 13 KB)

## Data Availability

The complete genomic nucleotide sequence of Phytophthora heveae alphaendornavirus 1 (PhAEV1) has been deposited in the NCBI GenBank database under accession number OQ786863. The dataset generated by HTS of total RNA contains unpublished viral sequences and is therefore available from the corresponding author only upon reasonable request.
