# Supplementary material for: Sequence and phylogenetic analysis of a novel alphaendornavirus, the first virus described from the oomycete plant pathogen Phytophthora heveae
Source: Arch Virol. 2023 May 11;168(6):158. doi: 10.1007/s00705-023-05786-7 (PMC10175314; doi:10.1007/s00705-023-05786-7)
Supplement: Supplementary file 1 — Supplementary file1 (DOCX 24 KB) [file 705_2023_5786_MOESM1_ESM.docx]

Supplementary Material

**Supplementary Table 1**. *Phytophthora heveae* isolates sequenced in a pooled RNA sample.

| Isolate | Site and location | Altitude | Host species and ecosystem | GenBank accession numbers | | RT-PCR  (yes/no) |
| --- | --- | --- | --- | --- | --- | --- |
|  |  |  |  | ITS | cox1 |  |
| VN0494a | Ba Vì National park | 1026 m | *Castanopsis chinensis, Beilschmiedia fordii* (soil); lowland rainforest | NA | NA | yes |
| VN0529 | Ba Vì National park | 809 m | *Lithocarpus* sp*.* (soil); lowland rainforest | MN872753 | MN866088 | No (dead) |
| VN0669 | Bu Gia Map National park | 417 m | *Dipterocarpus alatus, Ailanthus triphysa, Hopea odorata, Dalbergia oliveri* (soil); lowland rainforest | NA | NA | yes |
| VN0730 | Duong Minh Chau; Tay Ninh | 37 m | *Hevea brasiliensis* (soil); plantation | NA | NA | yes |
| VN0739 | Long Thanh; Dong Nai | 36 m | *Hevea brasiliensis* (soil); plantation | NA | NA | yes |
| VN0741 | Long Thanh; Dong Nai | 36 m | *Hevea brasiliensis* (soil); plantation | NA | NA | yes |
| VN0787 | Cuc Phuong National Park | 392 m | *Saraca dives*, 2 trees (soil); lowland rainforest | MN872769 | MN866095 | yes |
| VN0789 | Bù Gia Mập National park | 416 m | *Dipterocarpus alatus*, *Ailanthus triphysa*, *Hopea odorata*, *Dalbergia oliveri* (soil); lowland rainforest | MN872770 | MN866096 | No (dead) |
| VN1033 | Con dao island, Vung Tau | NA | *Hopea odorata*, *Dipterocarpus alatus* (soil); lowland rainforest | NA | NA | yes |

NA = Not Available. Further information available in Jung, T., Scanu, B., Brasier, C. M., Webber, J., Milenković, I., Corcobado, T., Tomšovský, M., Pánek, M., Bakonyi, J., Maia, C., Bačová, A., Raco, M., Rees, H., Pérez-Sierra, A., Horta Jung, M. (2020). A survey in natural forest ecosystems of Vietnam reveals high diversity of both new and described *Phytophthora* taxa including *P. ramorum*. Forests 11:93. <https://doi.org/10.3390/f11010093>.

**Supplementary table 2.** Detailed information of virus specific primers used in sequence validation and confirmation of virus presence

| Primer Name | Position Minimum | Position Maximum | Length | Primer orientation | Primer Sequence 5’-3’ | GC (%) | Tm (°C) | Amplicon size (bp) |
| --- | --- | --- | --- | --- | --- | --- | --- | --- |
| PhAEV_1_5_end_RV | 184 | 206 | 23 | reverse | ACATGTCTCCAAAAGCACAATCG | 43.5 | 60.1 | 206 |
| PhAEV_1_set_1a_FW | 2888 | 2909 | 22 | forward | ACTTTCAATGAGGTGTGCAGTC | 45.5 | 59.1 | 731 |
| PhAEV_1_set_1a_RV | 3597 | 3618 | 22 | reverse | AACACGAAAGAAGACGCATCAC | 45.5 | 59.8 | 731 |
| PhAEV_1_set_2_FW | 4956 | 4979 | 24 | forward | TGAACCCAGAATTTATTGACGAGG | 41.7 | 59.1 | 1015 |
| PhAEV_1_set_2_RV | 5950 | 5970 | 21 | reverse | TCCACTCCTCGCACTTCAAAG | 52.4 | 60.3 | 1015 |
| PhAEV_1_set_2a_FW | 3796 | 3817 | 22 | forward | AATGTGCATCCTTATTGACGGT | 40.9 | 58.4 | 578 |
| PhAEV_1_set_2a_RV | 4349 | 4373 | 25 | reverse | CATTAGCAGATGTTATTAGTTCCAC | 36 | 56.1 | 578 |
| PhAEV_1_set_3_FW | 6453 | 6474 | 22 | forward | GAAAGATTCGGAGGTTCACGTC | 50 | 59.3 | 984 |
| PhAEV_1_set_3_RV | 7415 | 7436 | 22 | reverse | AGCTATCCCTGATAATCTTGCC | 45.5 | 57.4 | 984 |
| PhAEV_1_set_3a_FW | 4772 | 4793 | 22 | forward | GACAACTACTCTGTTTCGCCAG | 50 | 59.3 | 536 |
| PhAEV_1_set_3a_RV | 5283 | 5307 | 25 | reverse | TGACTATTAAGGTTGGACAGGAGAC | 44 | 59.8 | 536 |
| PhAEV_1_set_4_FW | 7088 | 7108 | 21 | forward | AGTATGGGGGCGACAGAGTAT | 52.4 | 60.1 | 897 |
| PhAEV_1_set_4_RV | 7963 | 7984 | 22 | reverse | CATCACAACACCAACATCAATG | 40.9 | 56.5 | 897 |
| PhAEV_1_set_4a_FW | 7,625 | 7,645 | 21 | forward | GGTACCCTCATTGCAGCTCTG | 57.1 | 60.7 | 609 |
| PhAEV_1_set_4a_RV | 8213 | 8233 | 21 | reverse | AGCGTTGTCAAAATGCTCCAC | 47.6 | 60 | 609 |
| PhAEV_1_set_5_FW | 9767 | 9788 | 22 | forward | TCAGAAGATTTGCGTCATTGTG | 40.9 | 57.6 | 794 |
| PhAEV_1_set_5_RV | 10539 | 10560 | 22 | reverse | TCACGGGATGGCTGCTTATTAC | 50 | 60.5 | 794 |
| PhAEV_1_set_6_FW | 12245 | 12266 | 22 | forward | TGATGGCATTTCTGGGTGATGA | 45.5 | 60.0 | 439 |
| PhAEV1_set_6_RV | 12663 | 12683 | 21 | reverse | AAACTCCCTACATGACTTTGC | 42.9 | 56.2 | 439 |
| PhAEV_1_3_end_FW | 12481 | 12502 | 22 | forward | GGCGGAAAGGGAAACAAAGATC | 50 | 60.1 | 340 |

**Sequence and phylogenetic analyses of a novel alphaendornavirus, the first virus described from the oomycete plant pathogen *Phytophthora heveae***

Milica Raco^1^*, Thomas Jung^1^, Marilia Horta Jung^1^, Nguyen Minh Chi^2^, Leticia Botella^1^, Nobuhiro Suzuki^3^

^1^Phytophthora Research Centre, Department of Forest Protection and Wildlife Management, Faculty of Forestry and Wood Technology, Mendel University in Brno, Brno, Czech Republic

^2^Forest Protection Research Centre, Vietnamese Academy of Forest Sciences, 10000 Hanoi, Vietnam

^3^Institute of Plant Science and Resources, Okayama University, Kurashiki 7100046, Japan

*Correspondence: [milica.raco@mendelu.cz](mailto:milica.raco@mendelu.cz) (milica.raco@gmail.com)
